# Clinic-based evaluation study of the diagnostic accuracy of a dual rapid test for the screening of HIV and syphilis in pregnant women in Nigeria

**DOI:** 10.1371/journal.pone.0198698

**Published:** 2018-07-10

**Authors:** Ijaodola Olugbenga, Oyelade Taiwo, Maura Laverty, Evelyn Ngige, Chukwuma Anyaike, Rasheed Bakare, Veronica Ogunleye, Brandy L. Peterson Maddox, Daniel R. Newman, Harriet D. Gliddon, Eugenia Ofondu, Stephen Nurse-Findlay, Melanie M. Taylor

**Affiliations:** 1 National AIDS and STIs Control Programme, Federal Ministry of Health Nigeria, FCT Abuja, Nigeria; 2 WHO Regional Office for Africa, Brazzaville, Congo; 3 Department of Reproductive Health and Research, World Health Organization, Geneva, Switzerland; 4 Department of Microbiology, University College Hospital Ibadan, Ibadan, Nigeria; 5 Division of STD Prevention, National Center for HIV, Viral Hepatitis, STD and TB Prevention, Centers for Disease Control and Prevention, Atlanta, Georgia, United States of America; 6 London Centre for Nanotechnology, University College London, London, United Kingdom; 7 Dermatology and Venereology Department, Federal Medical Center Owerri, Owerri, Nigeria; Academic Medical Centre, NETHERLANDS

## Abstract

**Background:**

Screening pregnant women for HIV and syphilis is recommended by WHO in order to reduce mother-to-child transmission. We evaluated the field performance, feasibility, and acceptability of a dual rapid diagnostic test (RDT) for HIV and syphilis test in antenatal clinic settings in Nigeria.

**Methods and findings:**

Participants were recruited at 12 antenatal clinic sites in three states of Nigeria. All consenting individuals were tested according to the national HIV testing algorithm, as well as a dual RDT, the SD BIOLINE HIV/Syphilis Duo Test (Alere, USA), in the clinic. To determine sensitivity, specificity and concordance, whole blood samples were obtained for repeat RDT performance in the laboratory, as well as reference tests for HIV and syphilis. Dual test acceptability and operational characteristics were assessed among participants and clinic staff.

The prevalence of HIV among the 4,551 enrollees was 3.0% (138/4551) using the national clinic-based HIV testing algorithm. Positive and negative percent agreement of the HIV component of the dual RDT were 100.0% (95% CI 99.7–100.0) and 99.9% (95% CI 99.7–100.0) respectively, when compared with the national rapid testing algorithm. The prevalence of syphilis, using TPHA as the reference test, was low at 0.09% (4/4550). The sensitivity of the syphilis component of the dual RDT could not be calculated as no positive results were observed for patients that were positive for syphilis by TPHA. Each of the only four TPHA-positive specimens had RPR titers of 1:1 (neat), indicative of non-active syphilis. The specificity of the syphilis component of the dual RDT was 99.9% (95% CI 99.8–100.0).

The dual RDT received favorable feasibility ratings among antenatal care clinic staff. Acceptability among study participants was high with most women reporting preference for rapid dual HIV/syphilis testing.

**Conclusions:**

The SD BIOLINE HIV/Syphilis Duo Test showed a high overall diagnostic accuracy for HIV and a high specificity for syphilis diagnosis in antenatal clinic settings. This study adds to a growing body of evidence that supports the clinic-based use of dual tests for HIV and syphilis among pregnant women.

## Introduction

The global burden of mother-to-child transmission of HIV and syphilis continues to disproportionately affect populations in low and middle-income countries and reflects access to quality antenatal care services that include HIV and syphilis testing and treatment. Global and regional strategies and initiatives have been launched for dual elimination of mother-to-child transmission (EMTCT) of HIV and syphilis [1] [2] [3] [4], eleven countries have been validated for elimination to date [1], and a number of tools have been developed available to accelerate this [5]. Early detection and timely intervention of pregnant women infected with HIV and/or syphilis are required services indicators for country validation of EMTCT [1].

HIV transmission from mother-to-child occurs in approximately 15–30% of untreated pregnancies [4]. In 2015, Nigeria had the greatest number of new HIV infections among children in the world—an estimated 41,000 [range 28,000–57,000]—roughly equivalent to the next eight highest incidence countries combined. There has been a limited decline in new pediatric HIV infections in Nigeria since 2009; 21% compared to the 60% average among the other Global Plan priority countries [6].

Untreated maternal syphilis results in adverse pregnancy outcomes due to congenital syphilis in over half of affected pregnancies and can lead to early fetal loss, premature birth, stillbirth, neonatal death, low birth weight, and infant complications from infection [7]. Syphilis diagnosis and treatment is one of the most cost-effective and feasible intervention in low-resource and low prevalence settings [8] but studies show that antenatal screening coverage is approximately 38% in sub-Saharan Africa [9]. In 2015, in Nigeria, only 13.7% of pregnant women nationally were screened for syphilis and of those infected (1.2%) only 61% were estimated to have received treatment [10]. With an annual birth rate of over seven million annually [11], the contribution of congenital syphilis to adverse birth outcomes in Nigeria may be high. The need for prompt diagnosis and appropriate treatment of all infected pregnant women cannot be over emphasized in the bid for the EMTCT of HIV and syphilis.

Presently in Nigeria, diagnosis of HIV infection and syphilis in pregnant women is primarily achieved using separate tests. This has been noted to be time consuming for both the client and healthcare providers and associated with significant loss to follow-up for treatment of syphilis-positive cases among antenatal clinic (ANC) attendees. While HIV testing coverage in ANCs is fairly high in Nigeria, syphilis testing coverage in pregnant women is very low [10] [12]. One of the causes for this is likely to be that HIV testing is free, while syphilis testing is not. In addition, when syphilis testing is done, same day results are not available and treatment is delayed. Further, pregnant women testing positive for syphilis frequently have to pay for the benzathine benzylpenicillin treatment and its administration as an intramuscular injection.

Nigeria is amongst the countries prioritized for dual elimination of mother to child transmission of HIV and congenital syphilis [12]. Dual rapid diagnostic tests (RDTs) with adequate levels of performance may improve the timely detection of and treatment of HIV and syphilis infections among pregnant women in developing countries, which will facilitate elimination of mother-to-child transmission of HIV and syphilis when employed in antenatal settings [12, 13]. Recently, dual RDTs, used at the point-of-care for simultaneously detecting antibodies to HIV and *Treponema pallidum*, the causative agent of syphilis (dual HIV & syphilis treponemal RDTs) using venous whole blood, serum/plasma, or finger-stick whole blood have been developed and are now commercially available [13] [14] [15]. To date, there are few data on the performance of these dual RDTs in ANC settings, although they have been evaluated in several laboratory-based and field-based studies and have shown encouraging sensitivities and specificities as compared with reference laboratory tests [15].

In 2014, Nigeria performed a laboratory-based performance study of three dual HIV/syphilis RDT kits [12]. Subsequent to the laboratory evaluation of commercially available dual RDTs for HIV and syphilis, a donation of the SD BIOLINE HIV/Syphilis Duo RDT by Alere (USA) was made to the National Ministry of Health through the Organization of African First Ladies against AIDS (OAFLA). Nigeria chose this opportunity to evaluate the performance of this WHO-prequalified test kit as well as its feasibility and acceptability from the perspective of both the client and the healthcare worker. Twelve ANCs in three states in Nigeria were identified as study sites for this evaluation.

The primary objective of this study was to determine the diagnostic accuracy performance of the SD BIOLINE HIV/Syphilis Duo Test (the ‘dual RDT’) for the screening of HIV and syphilis in pregnant women compared to that of (1) the national HIV testing algorithm and (2) a laboratory-based 4^th^ generation HIV Enzyme Immunoassay (EIA) as two reference standards for HIV, and the *T*. *pallidum* Hemagglutination assay (TPHA) as the syphilis reference standard. The national HIV testing algorithm currently relies on HIV diagnosis using the Determine^TM^ HIV-1/2 Test (Alere), confirmed by Uni-Gold HIV Rapid Test (Trinity Biotech, Wicklow, Ireland). The 4^th^ generation HIV EIA used was the Genscreen Ultra HIV Ag-Ab Assay (BIO-RAD Europe GmbH). Since it detects HIV antigens as well as antibodies, it is more sensitive than tests that detect HIV antibodies alone, as is the case for Determine HIV-1/2 Test and the dual RDT, particularly in the acute stages of infection.

The secondary objective was to assess the acceptability and operational characteristics of the dual test among pregnant women and antenatal care clinic staff.

## Methods

### Ethics statement

Study protocol and consent was approved by WHO Ethics Research Committee (3^rd^ February 2017; protocol ID: A65909) and the National Health Research Ethics Committee of Nigeria (15^th^ November 2016; assigned number: NHREC/01/01/2007) prior to study initiation. All participants provided written informed consent for the diagnostic accuracy study and the operational characteristics questionnaire, and these were recorded and stored by the study team.

### Study site selection

A rapid assessment of facilities was conducted to assess and select suitable sites for this prospective study, and to identify study teams from each selected clinic facility. The selection criteria for the study sites included: (1) access to a sufficiently large population of pregnant women attending their first ANC visit in order to complete patient recruitment within 12 weeks of study initiation; (2) close proximity to the reference laboratory so that whole blood for reference tests could be processed and stored within eight hours of collection; (3) estimated prevalence of HIV and syphilis prevalence sufficiently high to achieve study parameters for performance analysis. Three reference laboratories and 12 enrollment sites (S1 Table) were selected in three states (Oyo, Imo and the Federal Capitol Territory (FCT)) in Nigeria. The sites chosen were healthcare facilities providing ANC services including HIV and syphilis testing.

The selection criteria for the reference laboratories selected included: (1) routine availability of reference testing for HIV and syphilis; (2) evidence of ongoing accreditation for laboratory quality assurance and management systems; (3) sufficiently trained laboratory and site staff with capacity to perform the study in accordance with the study protocol; (4) strong interest and commitment to research. Study teams consisted of nurses, physicians, laboratory scientists, phlebotomists, and record officers. Supervisory level staff included the state and site coordinator, data managers and reference laboratory coordinators.

### Study participants: Inclusion and exclusion criteria

Eligible participants included pregnant women presenting for their first ANC visit at one of the 12 study sites. Women were excluded if they were less than 18 years of age or could not provide informed consent.

### Pre-enrollment study activities and staff training

Study training was conducted over two seven-hour training sessions that took place over two consecutive days in each of the three States, FCT, Oyo and Imo, prior to study initiation. This included training on informed consent, data collection forms, data entry, and required study sample collection. S1 Fig shows an example training agenda. All study staff attended these trainings (S2 Table).

Hands-on training was provided by the dual RDT manufacturing company to all study staff who would be performing the tests in the field and in the laboratory setting. At least five staff members from each facility were trained in the use of the dual RDT. These included the study coordinators, ANC nurses, the laboratory phlebotomists, and the record officers from each site. Based on a simple training module, a pictorial training chart, appropriate for the local cultural context, was developed and used to train on the method to perform the tests according to manufacturer guidelines and to records results accurately.

### Study materials

The 10,100 SD BIOLINE HIV/Syphilis Duo Tests (Alere, USA) used for this study were donated by the manufacturer to the Nigerian government on June 2, 2016 and contained an expiration date of May 13, 2017. No other financial support from manufacturers was received. Other study items and materials were obtained through WHO funding. This included consumables, data collection forms, and benzathine benzylpenicillin.

### Sample size calculations for operational performance assessment

The sample size calculation was based on the laboratory performance of the dual RDT compared to the reference standard and the estimated HIV and syphilis prevalence in Nigeria. A disease seroprevalence of 5% for both HIV and syphilis in pregnant women was assumed based on recent surveillance data (2015), an assumed higher risk of HIV and/or syphilis in pregnant women, and the low uptake of HIV and syphilis testing in ANCs in Nigeria. Using an estimated test sensitivity of 98%, an alpha value of 0.05 and a beta value of 80% (power), a sample size of 3,800 was estimated. Based on the impreciseness of the available prevalence rates, particularly for syphilis, the sample size was increased to 4,500 women.

### Participant enrollment

**Fig 1:**
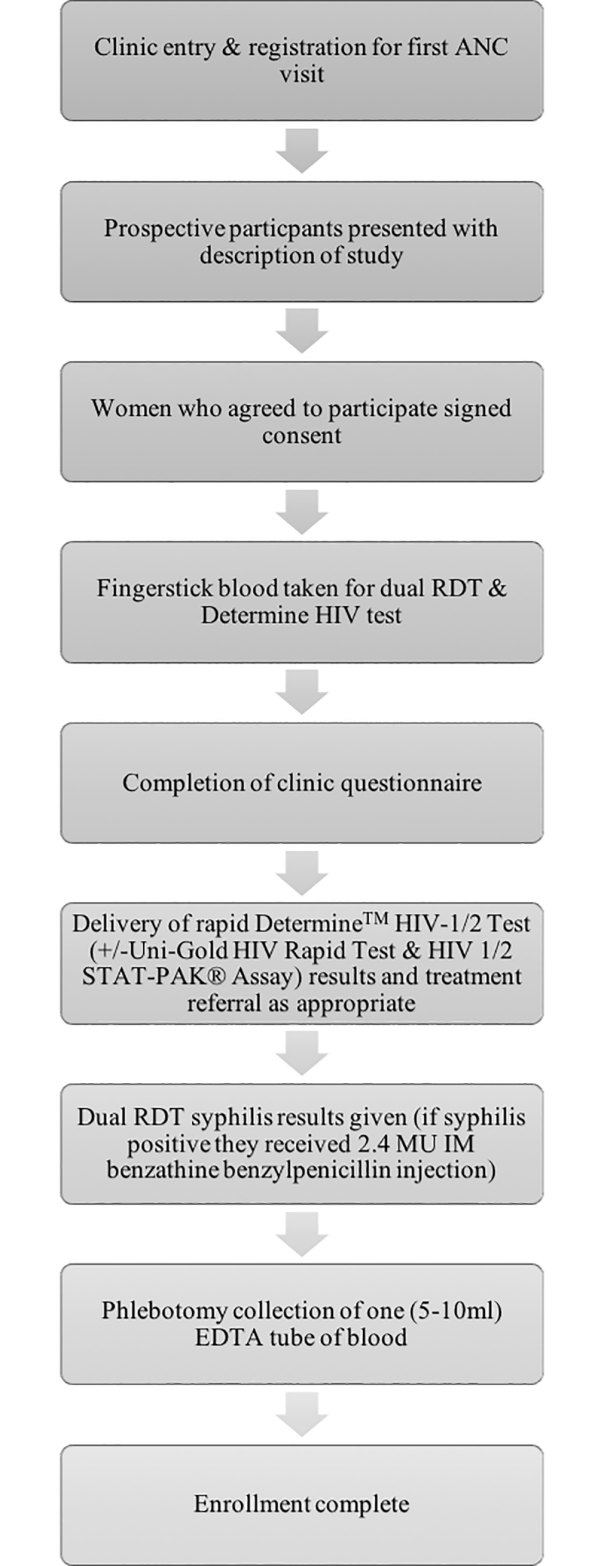
Recruitment and enrollment process of participants in ANC clinics.

Fig 1 describes the step-by-step enrollment, including informed consent and testing procedures used during the study to ensure uniformity across selected sites and consistency with the protocol. Roles and responsibilities of the study implementation team are described in S1 Table.

### Use of the dual RDTs and collection of specimens

All consenting ANC attendees were enrolled by a trained study nurse. The fingerprick blood sample obtained was used to perform the SD BIOLINE HIV/Syphilis Duo Test according to the manufacturer’s instructions, as well as the national HIV testing algorithm with Determine^TM^ HIV-1/2 Test, confirmed by Uni-Gold HIV Rapid Test. After 15–20 minutes, the nurse informed participants of the result of the Determine^TM^ HIV-1/2 Test and the syphilis result of the dual RDT and recorded all results on the socio-demographic questionnaire. In addition, 5ml of venipuncture whole blood was collected into an EDTA collection tube which was stored and then transported to the reference laboratory, in a temperature-controlled container (15°C) within eight hours of specimen collection.

**Fig 2:**
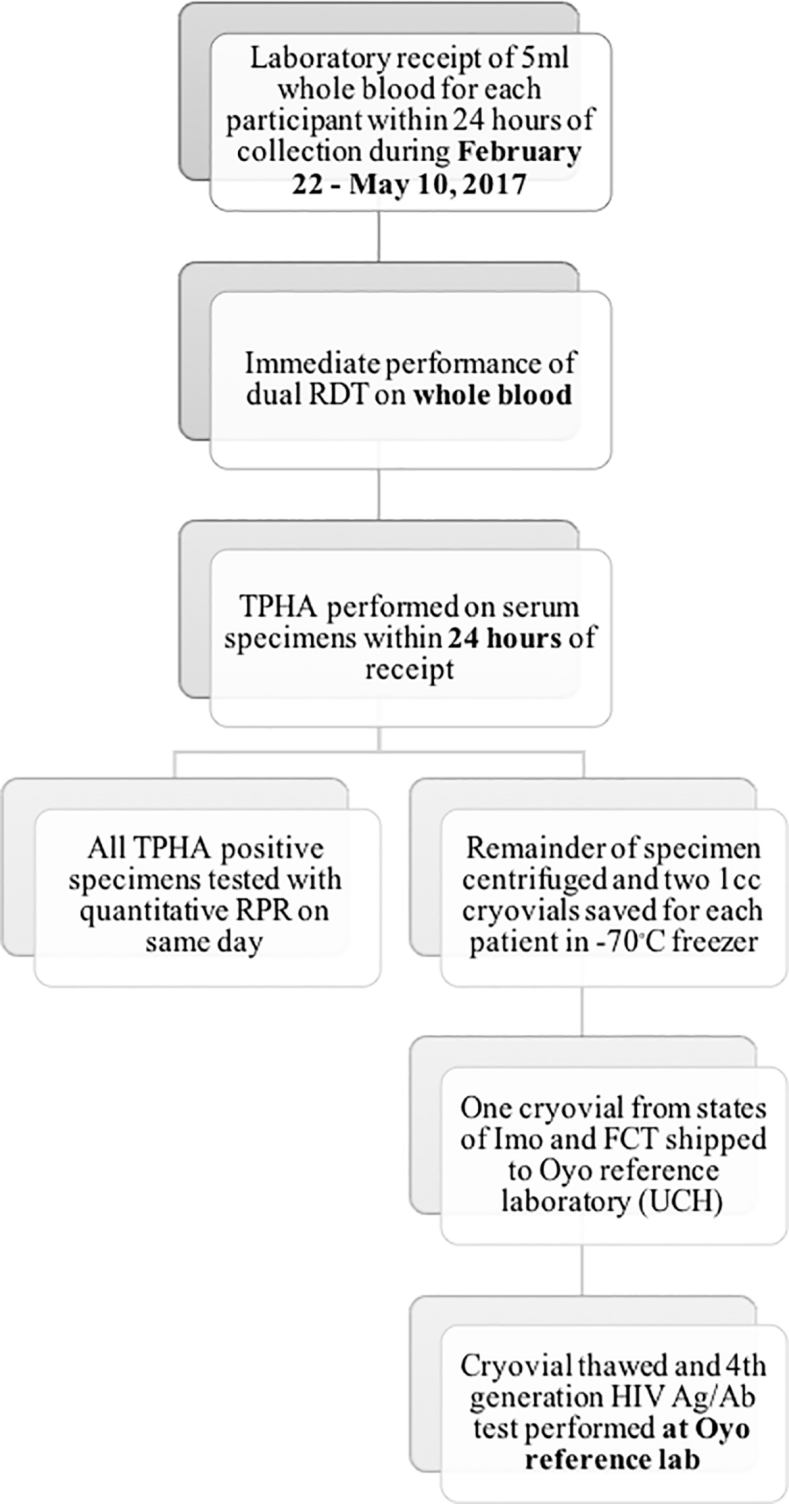
Workflow used for laboratory processing of specimens.

Upon arrival at the reference laboratory, a repeat dual RDT was performed on the whole blood specimen, by the laboratory staff, who were blinded to prior test results and clinic information of the participants (Fig 2). Study samples were then processed and stored as per manufacturer instructions for each of the tests performed in the laboratory. Two (2ml) aliquots of plasma were pipetted into cryovials and stored in a -70°C freezer. For syphilis reference testing, fresh whole blood specimens were tested with the TPHA. Those specimens that tested positive by TPHA were then tested using a quantitative RPR and the titer was recorded. The time interval between conducting the TPHA and the RPR, if required, on refrigerated samples was a maximum of one week. HIV reference tests were performed using stored samples at the end of the study using the Genscreen Ultra HIV Ag-Ab Assay, a 4^th^ generation HIV test. Frozen cryovials were shipped on dry ice from Imo and FCT states to the reference lab in Oyo state for performance of the HIV reference test. The time interval between the field-performed HIV tests and the batch testing of the Genscreen Ultra HIV Ag-Ab Assay was up to four months as serum was stored (-70°C) and batch tested at the end of the study. However, for a given participant, all reference samples in the study were taken at the same time as the field-performed dual RDT. All test results were recorded in a laboratory log.

### Data collection for feasibility and acceptability analysis

As part of the enrollment process, the consenting women completed the 10-item Socio-Demographic Participant Questionnaire (S2 Fig) to determine acceptability of the investigational dual RDT. Acceptability among participants was determined by asking the women if they would be willing to wait for their test results and if so, how long they would be willing to wait for these results (i.e., 30 minutes, one hour or two hours). Participants were also asked if they preferred a single test or dual test for HIV and syphilis.

Assessment of the feasibility of the dual RDT was conducted among ANC staff members. A brief six-item Dual Test Operational Characteristics Questionnaire (S3 Fig) was administered to ANC clinic staff (i.e. providers, nurses, laboratory technicians) from each field evaluation site, who performed and read the test, to determine operational characteristics by rating six test attributes (e.g. clarity of instructions, ease of use, and readability of test results). ANC clinic staff completed the operational characteristics questionnaire at the end of participant enrollment. Consent to complete the operational characteristics questionnaire was obtained prior to providing the survey to ANC staff. Study site coordinators were responsible for collecting and managing the participant and ANC staff questionnaires.

### Feasibility and acceptability data analysis

CDC DSTDP staff led the data analysis and did not have contact with human subjects for this project component. Microsoft Excel was used for initial data management and quantitative data analysis was conducted using SAS (Version 9.4). Quantitative data were principally analyzed using descriptive statistics. Data from staff and participant questionnaires were reviewed for dual test preferences and other pertinent test acceptability information. Proportions were used to categorize and summarize the baseline characteristics.

Analysis of acceptability of the dual RDT was performed using the proportion of pregnant women who preferred dual RDT test as well as the proportion willing to wait for test results and for how long. To assess the operational feasibility of the dual RDT, ANC clinic staff rated test attributes (i.e., minimum operational characteristic) on a scale of 0–2 (2 attributes) or 0–3 (4 attributes) and we calculated the mean score for all staff who responded to the questionnaire for each attribute. Attributes scaled 0–2 with a score ≥ 1.50 and scaled 0–3 with a score ≥ 2.25 were coded acceptable. An overall feasibility score was calculated as the total of the mean attribute scores.

### Diagnostic test accuracy statistical analysis

The sensitivity and specificity of the dual RDT was assessed using two separate reference standards for HIV; (1) the national rapid testing algorithm with Determine^TM^ HIV-1/2 Test (Alere) positivity confirmed by a positive Uni-Gold HIV Rapid Test (Trinity Biotech) and (2) the laboratory-based HIV 4^th^ generation Ag/Ab Enzyme Immunoassay (EIA) (Genscreen ULTRA HIV Ag-Ab Assay). This allowed a comparison between the dual RDT and the current HIV testing standard in ANC settings in the field, and also a gold standard laboratory-based assay. The accuracy of the syphilis component was assessed using the TPHA (Human Gesellschaft fur Biochemica und Diagnostica mbH, Wiesbaden, Germany) as a laboratory reference standard for antibodies to *T*. *pallidum*. A rapid plasma reagin (RPR) assay (Syphilis RPR test, Human Gesellschaft fur Biochemica und Diagnostica mbH, Wiesbaden, Germany) was conducted for samples that gave a positive TPHA result in order to identify acute infection (RPR≥ 1:4).

The sensitivity, specificity and kappa statistics [16] were calculated using the EpiBasix package [17] in R statistics (version 3.4.0) [18]. 95% confidence intervals were calculated using the exact binomial method.

## Results

### Study population

Table 1 lists the characteristics of the 4,551 study participants recruited across the 12 selected study sites. The median age of the women recruited to the study was 30 years (Interquartile range (IQR): 27–33). The median gestational age was 20 weeks (IQR: 16–28) and the median time (minutes) taken to reach the clinic from their homes was 30 minutes (IQR: 30–50).

**Table 1:** Characteristics of study participants, including their age, gestation length and the time required to reach the ANC clinic from home.

	Age of mother (years)	Estimated length of gestation (weeks)	Time taken to reach clinic from home (minutes)
**Median (IQR)**	30 (27–33)	20 (16–28)	30 (30–50)

### Specimen collection and reporting of results

All participants had a dual RDT, Determine HIV-1/2 Test (Alere) (as well as a Uni-Gold™ HIV Rapid Test (Trinity Biotech) for confirmation of a positive result, and a HIV 1/2 STAT-PAK® Assay (Chembio) test upon a negative Uni-Gold™ HIV Rapid Test result) at the ANC clinic. Participants received HIV results from the national HIV testing algorithm and the syphilis component of the RDT. A completed STARD checklist and all diagnostic test accuracy results can be found in the supplementary information (S1 File and S2 File respectively).

Serum samples used for laboratory testing with the dual RDT and reference tests for antibodies to HIV and syphilis were collected from 4,550 participants. One participant experienced failed attempts at blood draw and was therefore unable to be tested using the HIV EIA reference testing. However, rapid test results for the dual RDT and Determine^TM^ HIV-1/2 Test/Uni-Gold™ HIV Rapid Test were available from the ANC setting. Therefore, this individual could only be included in the analysis comparing the accuracy of the HIV component of the dual RDT to Determine^TM^ HIV-1/2 Test/Uni-Gold™ HIV Rapid Test. There were no other missing results, and no indeterminate or inconclusive results were given by any of the RDTs or laboratory assays used in the study.

Four participants tested positive for syphilis using the dual RDT, but negative when the TPHA was performed in the laboratory. For two of these participants, the dual RDT was repeated on the advice of senior clinical team during the same clinic visit with subsequent negative result for syphilis. Therefore, these two participants were not treated for syphilis. The false positive results were probably caused by incorrect interpretation of the dual RDT. Two participants received benzathine benzylpenicillin due to a positive syphilis dual RDT result, but subsequently had a negative TPHA result. This minor overtreatment is the only adverse effect of the study that the authors are aware of.

### Prevalence of HIV and syphilis

Prevalence of HIV among study participants was 3.0% (138/4551) using the national clinic-based point-of-care HIV testing algorithm. Prevalence of TPHA positivity for syphilis was low at 0.09% (4/4550). Of the four TPHA positive results, all were positive by RPR only at a titer of 1:1, likely indicating previously treated infection.

### HIV clinical sensitivity and specificity of the dual RDT in the field

#### HIV: Sensitivity and specificity of the dual RDT (field-performed) versus 4^th^ generation HIV Ag/Ab laboratory reference (Table 2)

Compared to the laboratory-based 4^th^ generation HIV Ag/Ab reference test, the performance of the HIV component of the dual RDT had a sensitivity of 85.8% (95% CI 79.1–90.6) and a specificity of 99.5% (95% CI 99.3–99.7) with a Kappa coefficient of 0.847 (95% CI 0.802–0.893).

**Table 2:** Summary of diagnostic accuracy results.

Index test	Reference test	TP	FN	FP	TN	Total	Sensitivity (%) (95% CI)	Specificity (%) (95% CI)	Kappa Coefficient (95% CI)
**SD BIOLINE HIV/Syphilis Duo Test (HIV)**	Genscreen ULTRA HIV Ag-Ab Assay	121	20	22	4387	4550	85.8 (79.1–90.6)	99.5 (99.3–99.7)	0.847 (0.802–0.893)
**Determine^TM^ HIV-1/2 Test (+/-Uni-Gold HIV Rapid Test & HIV 1/2 STAT-PAK® Assay)**	Genscreen ULTRA HIV Ag-Ab Assay	115	26	22	4387	4550	81.6 (74.4–87.1)	99.5 (99.3–99.7)	0.822 (0.772–0.871)
**SD BIOLINE HIV/Syphilis Duo Test (HIV)**	Determine^TM^ HIV-1/2 Test (+/-Uni-Gold HIV Rapid Test & HIV 1/2 STAT-PAK® Assay)	138	0	6	4407	4551	100.0 (97.3–100.0)*	99.9 (99.7–100.0)*	0.978 (0.960–0.996)
**SD BIOLINE HIV/Syphilis Duo Test (syphilis)**	Syphilis TPHA	0	4	4	4542	4550	N/A	99.9 (99.8–100.0)	N/A

Results are shown for both the HIV and syphilis components of the SD BIOLINE HIV/Syphilis Duo Test, and the national rapid testing protocol (Determine^TM^ HIV-1/2 Test, +/- Uni-Gold HIV Rapid Test & HIV 1/2 STAT-PAK® Assay) (TP: true positives; FN: false negatives; FP: false positives; TN: true negatives; CI: confidence intervals).

*Where the accuracy of the dual RDT was compared to the non-standard reference test Determine^TM^ HIV-1/2 Test (+/-Uni-Gold HIV Rapid Test & HIV 1/2 STAT-PAK® Assay), the sensitivity and specificity should be substituted for positive and negative percent agreement.

#### HIV: Nigeria national HIV testing algorithm (field-based Determine^TM^ HIV-1/2 Test with Uni-Gold™ HIV Rapid Test confirmation) versus 4^th^ generation HIV Ag/Ab laboratory reference (Table 2)

Compared to the laboratory-based 4^th^ generation HIV Ag/Ab reference test, the performance of the Nigeria national HIV testing algorithm included a sensitivity of 81.6% (95% CI 74.4–87.1) and specificity of 99.5% (95% CI 99.3–99.7) with Kappa coefficient of 0.822 (95% CI 0.772–0.871).

#### HIV: Dual RDT (field-performed) versus Nigeria national HIV testing algorithm (field-based Determine^TM^ HIV-1/2 Test with Uni-Gold™ HIV Rapid Test confirmation) (Table 2)

Although Nigeria’s national HIV testing algorithm is not a standard reference test for HIV diagnosis, it is nevertheless a useful comparison for the dual RDT. Where a non-standard reference test is used, sensitivity and specificty should be substituted for positive percent agreement and negative percent agreement, respectively. The positive percent agreement of the HIV component of the dual RDT was 100.0% (95% CI 99.7–100.0) compared with the national testing algorithm (clinic-based Determine^TM^ HIV-1/2 Test /Uni-Gold™ HIV Rapid Test) and negative percent agreement was 99.9% (95% CI 99.7–100.0) with a Kappa co-efficient of 0.978 (95% CI 0.960–0.996).

### Syphilis clinical sensitivity and specificity of the SD BIOLINE HIV/Syphilis Duo test in the field

#### Comparison of the syphilis component of the dual RDT with laboratory-based TPHA (Table 2)

The sensitivity of the syphilis component of the dual RDT could not be calculated because there were no positive results for both the TPHA and the dual RDT. Four specimens out of the 4,550 tested were TPHA positive, but these were not identified as positive using the dual RDT. Each of these four specimens were positive by non-treponemal RPR testing but were reported as having a titer of 1:1 (neat), indicative of non-active syphilis.

The specificity of syphilis diagnosis using the dual RDT was 99.9% (95% CI 99.8–100.0), using TPHA as the laboratory reference standard for syphilis infection. There were four false positives, where a positive syphilis result was recorded for the dual RDT, but the TPHA test was subsequently found to be negative. Two of these occurred at the State Hospital Adeoyo (Oyo State) and were the only positive syphilis results seen at this site using the dual RDT. These two positive results occurred on the same day and within the first two weeks of the study, and they coincided on both occasions with a positive HIV result. It is possible that these two tests were mistakenly interpreted as positive for both HIV and syphilis rather than positive for HIV alone. Same day repeat testing with the RDT yielded negative syphilis results for these two participants.

### Concordance of the dual RDT when conducted in laboratory and field settings (Table 3)

A high concordance between laboratory and field settings was observed for HIV diagnosis, with a kappa value of 0.967 (CI_95%_ 0.946–0.989). However, the concordance for syphilis was lower, with a kappa value of 0.666 (CI_95%_ 0.358–0.974).

**Table 3:** Concordance for HIV and syphilis diagnosis by the SD BIOLINE HIV/Syphilis Duo test when conducted in laboratory and field settings.

Concordance analysis	TP	FN	FP	TN	Total	Kappa Coefficient (95% CI)
**HIV: Field vs. laboratory settings**	137	3	6	4404	4550	0.967 (0.946–0.989)
**Syphilis: Field vs. laboratory settings**	4	1	3	4542	4550	0.666 (0.358–0.974)

TP: true positives; FN: false negatives; FP: false positives; TN: true negatives; CI: confidence intervals

### Dual RDT acceptability among participants

Acceptability data were obtained from the 4,551 pregnant women enrolled in the study. As displayed in Table 4, the level of acceptability of the dual RDT was relatively the same among all sites and pregnant women overwhelmingly reported a preference for the dual HIV/syphilis RDT (99.9%) compared to single tests for HIV and syphilis (0.1%). Only five women preferred a single test (0.1%) and one women stated the reason was because she wanted, ‘to be sure of different results’. Nearly all women were willing to wait at the clinic for their test results (99.9%). About three-quarters (73.8%) were willing to wait up to 30 minutes.

**Table 4:** Acceptability of dual RDT among pregnant women attending antenatal care in Nigeria by baseline characteristics.

Characteristics	Number (%) of test preference				Number (%) willing to wait for results	% of those willing to wait for:		
	N	Dual	Single	Don’t know/don’t care		30mins	1hr	2hrs
**Age (Years)**								
15–19	63	63 (100.0)	0 (0.0)	0 (0.0)	63 (1.4)	76.2	19.1	4.8
20–24	588	586 (99.6)	2 (0.34)	0 (0.0)	587 (12.9)	69.2	18.7	12.1
25–29	1514	1510 (99.7)	3 (0.2)	1 (0.1)	1513 (33.3)	73.8	14.9	11.3
30–34	1538	1537 (99.9)	0 (0.0)	1 (0.1)	1538 (33.8)	74.4	14.0	11.6
35–45	841	841 (100.0)	0 (0.0)	0 (0.0)	841 (18.5)	75.5	15.2	9.3
46–55	6	6 (100.0)	0 (0.0)	0 (0.0)	6 (0.1)	66.7	16.7	16.7
**Gestational age (Weeks)**								
1–12	689	687 (99.7)	2 (0.3)	0 (0.0)	688 (15.1)	72.5	14.1	13.4
13–24	2490	2487 (99.9)	2 (0.1)	1 (0.0)	2489 (54.74)	71.8	15.4	12.8
25+	1370	1368 (99.9)	1 (0.1)	1 (0.1)	1370 (30.1)	77.9	15.3	6.8
Missing	2				4			
**Gestational age (Weeks #2)**								
<20	1713	1711 (99.9)	2 (0.1)	0 (0.0)	1713 (37.7)	73.5	14.4	2.1
20+	2836	2831 (99.8)	3 (0.1)	2 (0.1)	2834 (62.3)	73.9	15.7	10.4
Missing	2				4			
**Travel time (mins)**								
≤30mins	2417	2412 (99.8)	4 (0.2)	1 (0.0)	2416 (99.9)	74.6	15.8	9.6
31–60mins	1970	1969 (99.9)	0 (0.0)	1 (0.1)	1970 (100.0)	73.2	14.3	12.4
≥61mins	160	159 (99.3)	1 (0.6)	0 (0.0)	159 (99.4)	67.3	17.0	15.7
Missing	4				6			
**Clinic site**								
FCT-Abuja	1511	1509 (99.9)	2 (0.1)	0 (0.0)	1509 (33.2)	51.4	19.0	29.6
Oyo-Ibadan	1540	1537 (99.8)	2 (0.1)	1 (0.1)	1540 (33.7)	80.8	16.7	2.5
Imo-Owerri	1499	1497 (99.9)	1 (0.1)	1 (0.1)	1499 (33.0)	89.0	9.8	1.2
Missing	1				3			
**Total**	4551	4543 (99.9)	5 (0.1)	2 (0.0)	4549 (99.9)	73.8	15.2	11.1

### Dual RDT acceptability and operational characteristics among ANC staff

Forty-eight ANC clinic staff completed surveys on the dual RDT’s minimum operational characteristics. The ANC staff characteristics are displayed in S3 Table. Completed surveys among clinic staff was relatively proportional across the three states, with the majority of responses from site nurses (50.0%) who had experience directly using the test as part of the study.

Regarding operational feasibility, the ANC clinic staff rated the dual RDT with an overall feasibility score of 12.3 out of a maximum obtainable score of 16 (Table 5). Clinic staff in Oyo State rated feasibility of the test higher than clinic staff in FCT and Imo State (13.1, 12.5, and 11.1, respectively). Overall, ANC staff provided an acceptable rating for 4 of 6 operational attributes, with the exception of ‘hands-on time’ (scale: 0–2, score: 1.35) and ‘training time required’ (scale: 0–3, score: 1.95). All results for the acceptability and operational characteristics of the dual RDT are listed in the supplementary information (S3 File).

**Table 5:** Operational feasibility of dual HIV-syphilis rapid diagnostic test among ANC clinic staff by site.

Operational characteristics	Mean Score (SD)			
	FCT-Abuja n = 16	OYO-Ibadan n = 17	IMO-Owerri n = 15	All sites n = 48
**Clarity of kit instruction (MOS = 3)**	2.43 (0.51)	2.52 (0.71)	2.20 (0.56)	2.39 (0.60)
**Ease of use (MOS = 3)**	2.56 (0.51)	2.41 (0.50)	2.26 (0.59)	2.41 (0.53)
**Ease of interpretation of results (MOS = 3)**	2.18 (0.40)	2.58 (0.50)	2.00 (0.53)	2.27 (0.53)
**Rapidity of test results (MOS = 2)**	2.00 (0)	1.91 (0.24)	1.80 (0.41)	1.91 (0.27)
**Hands-on time (MOS = 2)**	1.25 (0.77)	1.64 (0.60)	1.13 (0.63)	1.35 (0.69)
**Training time required (MOS = 3)**	2.06 (0.77)	2.05 (0.89)	1.73 (1.22)	1.95 (0.96)
**Total (MOS = 16)**	12.5	13.1	11.1	12.3
*MOS = Maximum Obtainable Score*				

## Discussion

This study is one of the few to assess field performance characteristics and acceptability of a novel, dual RDT for HIV and syphilis. Our findings are in line with previous studies, demonstrating high accuracy for diagnosis of HIV. Low prevalence of syphilis limited the ability to fully assess performance of this component of the test. The findings from the assessment of the acceptability and minimum operational characteristics of a dual HIV/syphilis RDT reflect similar findings in other studies assessing dual RDT acceptability and feasibility. The strength of this assessment is the ability to document test performance as well as perceptions of both pregnant women, the population likely to benefit from dual RDT use, and ANC staff with experience using the dual RDT in the clinic setting.

The positive percent agreement of the HIV component of the dual RDT compared to the national HIV testing algorithm (Determine^TM^ HIV-1/2 Test, +/- Uni-Gold™ HIV Rapid Test & HIV 1/2 STAT-PAK® Assay) was 100.0%; specificity was likewise high (99.9%). The sensitivities of both the dual RDT and the Determine^TM^ HIV-1/2 Test (3^rd^ generation HIV EIA assays) were lower when compared to the 4^th^ generation Ag-Ab test, a result to be expected due to the different generation of HIV EIA assays. We were unable to elucidate whether the additional detection of p24 by the 4^th^ generation reference test explains the observed low sensitivity of the rapid tests, because the Genscreen ULTRA HIV Ag-Ab Assay test does not provide separate results for HIV-1/2 antigens and HIV-1/2 antibodies. Although the sensitivity of the dual RDT was higher for HIV diagnosis than the national HIV testing algorithm, this difference was not statistically significant, as the confidence intervals for each of the sensitivities overlapped. The prevalence of syphilis was too low to calculate the sensitivity of the syphilis component of the dual RDT but specificity was high (99.9%). Of the four participants that tested positive via the syphilis TPHA laboratory reference test (all four RDT syphilis-negative) all had low-titer RPR values of 1:1 likely reflecting old treated infection. Previous studies have shown a reduced sensitivity of dual RDTs for syphilis cases where the RPR titer is low [19, 20]. This has significant implications for training with the use of dual RDTs, since positive test responses for HIV and syphilis have been shown to appear with differing levels of band color intensity, with bands of darker intensity observed for HIV than syphilis [21, 22]. The concordance of the laboratory performance versus field-performance of the HIV component of the RDT was high (Kappa 0.967) however, for syphilis the concordance of the RDT was lower (Kappa 0.666) likely due to training issues regarding the syphilis test result interpretation and limited experience with use of this RDT.

Patient acceptability of the dual RDT was positive. Almost all women preferred the dual RDT and the majority were willing to wait up to 30 minutes for their test results. Nearly half of the women noted experiencing travel times to the clinic of greater than 30 minutes. Their willingness to wait up to 30 minutes for results of the dual RDT may be a more favorable choice than having to return to the clinic at a later time for syphilis results and treatment. The dual RDT received favorable feasibility ratings among antenatal care clinic staff with experience using the test in a clinic-based setting. Assessing the acceptability and minimum operational characteristics of this dual HIV/syphilis RDT provides timely and useful information beyond test performance for the Nigerian Ministry of Health (MoH) in consideration of use and scale-up use of the test across the country.

There are limitations that should be considered regarding the interpretation of these data. First, this was a convenience sample of pregnant women attending their first ANC visit limiting representativeness of pregnant women in Nigeria overall. Participant enrollment was limited to a specific time period reflecting the expiration dates of the donated test kits. The sensitivity of the syphilis component of the dual RDT was a key expected result from the study. However, a low prevalence of syphilis in this large study size of 4,551 pregnant women was unanticipated and prevented this calculation. This low syphilis prevalence is not representative of recent syphilis prevalence survey estimates in Nigeria. External quality assurance of HIV and syphilis testing was not performed through an outside reference lab. However, each of the reference laboratories were nationally accredited. Finally, use of the 4^th^ generation HIV Ag/Ag test (Genscreen ULTRA HIV Ag-Ab Assay) as the laboratory reference standard for HIV testing did not reflect a true comparison with the HIV EIA component of the dual RDT. The ability of the 4^th^ generation HIV to detect acute HIV infection prior to antibody seroconversion was reflected in the calculated sensitivities for the dual RDT test as well as the national rapid HIV testing algorithm which uses Determine^TM^ HIV-1/2 Test as the initial screening test.

The results from this assessment are being used by the Nigerian MoH to facilitate initial decision-making for future use and potential scale-up of the dual test in ANC settings. As the Nigerian MoH reviews these favorable outcomes, the next step potentially is how this information will be used to inform national policies, HIV and syphilis testing algorithms, and clinic practices of the sites included in the evaluation. An in-depth qualitative assessment may help the MoH identify ways to address barriers and implement strategies to improve acceptability and feasibility of dual HIV/syphilis testing provision and develop a plan of action to address key barriers.

Ministries of Health, outside of Nigeria, considering a similar implementation may be interested in the evaluation findings and this approach to validate test performance and assess acceptability, feasibility and dual testing barriers and facilitators within their own countries. This study adds to a growing body of evidence that supports the clinic-based use of dual tests for HIV and syphilis among pregnant women and can be used as further justification of their use in antenatal settings.

## Supporting information

S1 Table Study enrollment sites consisted of 12 ANCs in three states in Nigeria. (PDF)

S2 Table Study personnel and site roles and responsibilities. (PDF)

S3 Table Characteristics of clinic staff assessing dual test operational characteristics. (PDF)

S1 Fig Example agenda for pilot training. (PNG)

S2 Fig Socio-demographic participant questionnaire used in the study. (PNG)

S3 Fig Dual test operational characteristics questionnaire used in the study. (PNG)

S1 File STARD checklist. (DOCX)

S2 File Diagnostic test accuracy data. (XLSX)

S3 File Dual RDT acceptability and operational characteristics data. (XLSX)
