# Correction: Clinic-based evaluation study of the diagnostic accuracy of a dual rapid test for the screening of HIV and syphilis in pregnant women in Nigeria

**DOI:** 10.1371/journal.pone.0202122

**Published:** 2018-08-06

**Authors:** The PLOS ONE Staff

The eleventh author's name is spelled incorrectly in the PDF version of this paper. The correct name is: Eugenia Ofondu. The publisher apologizes for the error.
