# Supplementary material for: Clinic-based evaluation study of the diagnostic accuracy of a dual rapid test for the screening of HIV and syphilis in pregnant women in Nigeria
Source: PLoS One. 2018 Jul 10;13(7):e0198698. doi: 10.1371/journal.pone.0198698 (PMC6038984; doi:10.1371/journal.pone.0198698)
Supplement: S1 Table — (PDF) [file pone.0198698.s001.pdf]

Table S1. Study enrollment sites consisted of 12 ANCs in three states in Nigeria.

State	ANCs
**Federal Capital Territory (FCT)**	Maitama District Hospital<br>Wuse District Hospital<br>Asokoro District Hospital<br>Primary Health Center, Mpape
**Ibadan**	State Hospital Adeoyo<br>Abongbon Primary Health Center<br>University College Hospital (UCH), Ibadan<br>Eleta Hospital
**Imo**	Federal Medical Center, Owerri<br>Specialist Hospital Owerri<br>St David’s Hospital, Owerri<br>Holy Rosary Hospital, Emekuku
