# Supplementary material for: Clinic-based evaluation study of the diagnostic accuracy of a dual rapid test for the screening of HIV and syphilis in pregnant women in Nigeria
Source: PLoS One. 2018 Jul 10;13(7):e0198698. doi: 10.1371/journal.pone.0198698 (PMC6038984; doi:10.1371/journal.pone.0198698)
Supplement: S2 Table — (PDF) [file pone.0198698.s002.pdf]

Table S2. Study personnel and site roles and responsibilities.

Study personnel description	Roles and responsibilities
**National/State-level coordination staff**	
**National Team Study Coordinator**	Coordinates and manages all study activities centrally including dissemination of positive reference laboratory test results to state coordinators and study clinics
**State Study Coordinator**	Overall manager and coordinator of the study at each of three state levels (3 state managers)
**State Data Entry Officer**	Ensures that the completed questionnaires and laboratory results are entered into the Excel database
**Clinic-Level Staff**	
**Site Study Coordinator-**	Coordinates all study activities at the ANC site level
**Study Nurse**	1. Informed consent<br>2. Administer clinical data collection form.<br>3. Perform SD BIOLINE Duo HIV/Syphilis Test on fingerstick blood<br>4. Retrieve the Determine^{TM} HIV-1/2 Test HIV fingerstick test<br>5. Record results
**Site Laboratory Liaison Officer**	Pick-up blood samples from site and bring to reference lab
**Clinic site phlebotomist**	Collect 5 ml of blood for the study in (2) EDTA tubes<br>1. Label each tube with PID, place sample in plastic bag with remaining PID stickers<br>2. Store room temperature until pick/up.
**Record Officers**	Ensures that the all study forms are completed and are entered into excel
**Reference Laboratory Staff**	
**Reference laboratory staff**	1. Receive and log in specimens<br>2. Conduct SD Bioline RDT on whole blood and record results<br>3. Centrifuge sample and save two 0.5mls Cryovials and store at -80 ^{O}C<br>4. Perform TPHA on every specimen<br>5. Perform RPR (quantitative) on TPHA positive samples<br>6. Perform HIV EIA on every specimen<br>7. Perform Western blot on all HIV EIA positives
