# Supplementary material for: Clinic-based evaluation study of the diagnostic accuracy of a dual rapid test for the screening of HIV and syphilis in pregnant women in Nigeria
Source: PLoS One. 2018 Jul 10;13(7):e0198698. doi: 10.1371/journal.pone.0198698 (PMC6038984; doi:10.1371/journal.pone.0198698)
Supplement: S3 Table — (PDF) [file pone.0198698.s003.pdf]

Table S3. Characteristics of clinic staff assessing dual test operational characteristics.

Characteristics	Number of staff	% of total
**Staff position**		
Site coordinator	11	22.9
Site nurse	24	50.0
Reference laboratorian	11	22.9
Phlebotomist	2	4.2
**Clinic site**		
FCT-Abuja	16	33.3
Oyo-Ibadan	17	35.4
IMO-Owerri	15	31.3
**Total**	48	100
