# Supplementary figures and images for: Clinic-based evaluation study of the diagnostic accuracy of a dual rapid test for the screening of HIV and syphilis in pregnant women in Nigeria
Source: PLoS One. 2018 Jul 10;13(7):e0198698. doi: 10.1371/journal.pone.0198698 (PMC6038984; doi:10.1371/journal.pone.0198698)

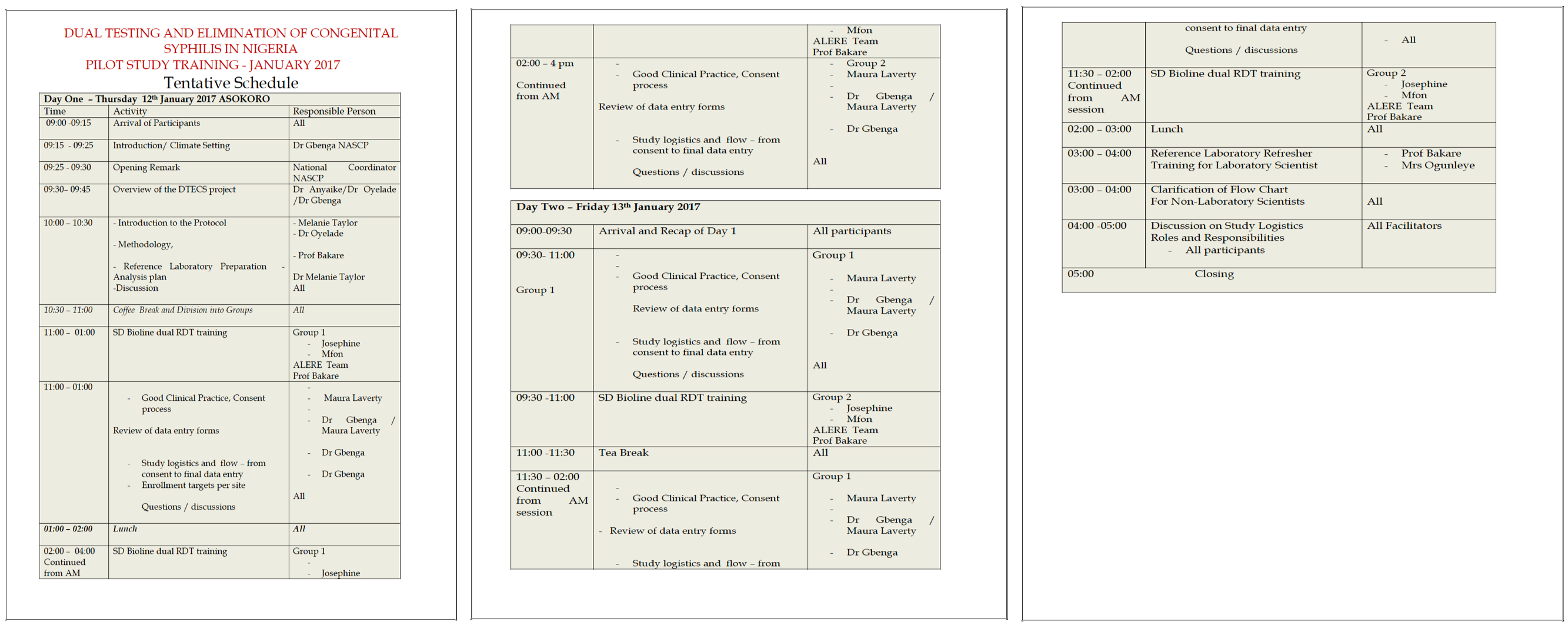

Supplement: S1 Fig — (PNG) [file pone.0198698.s004.png]

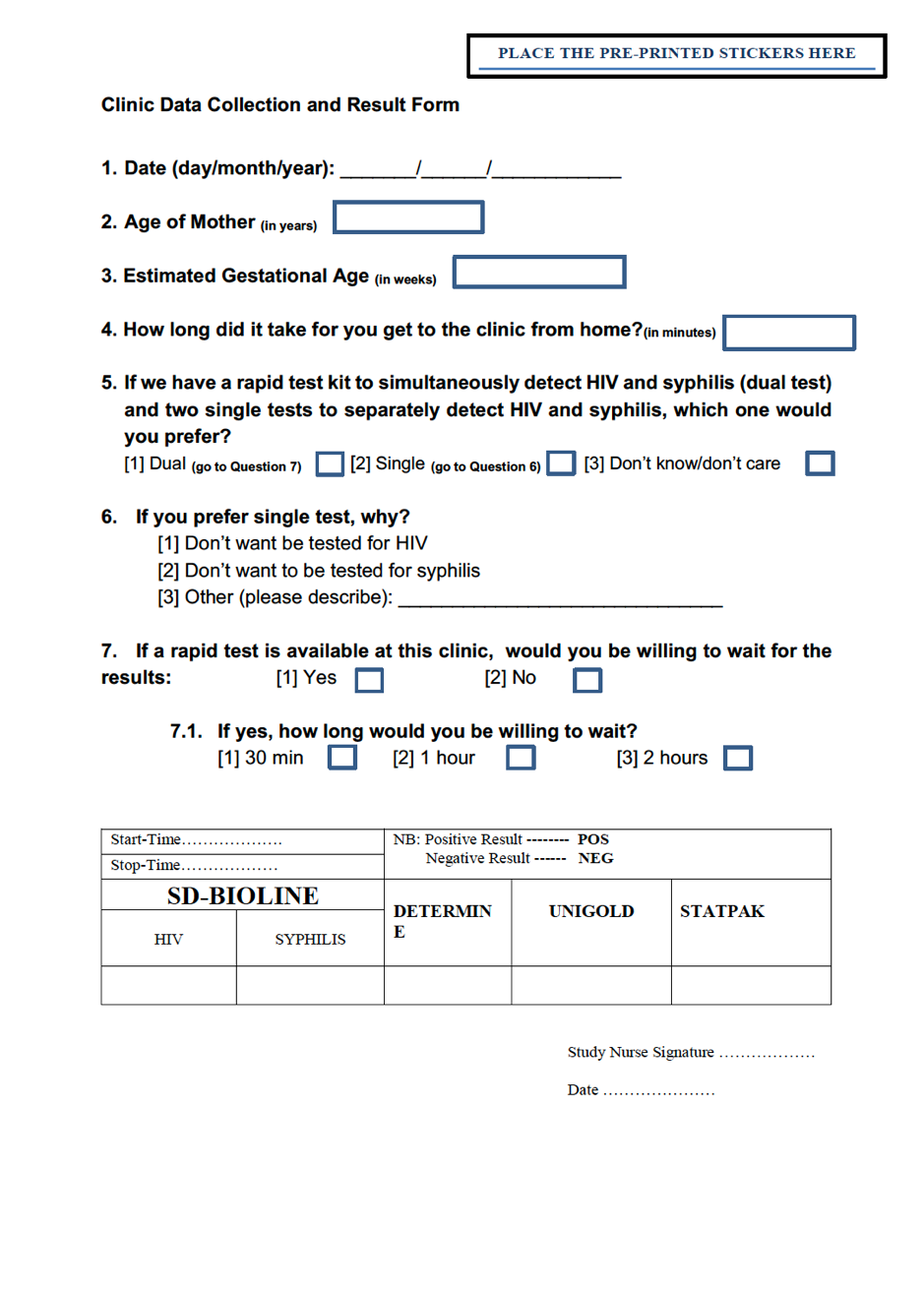

Supplement: S2 Fig — (PNG) [file pone.0198698.s005.png]

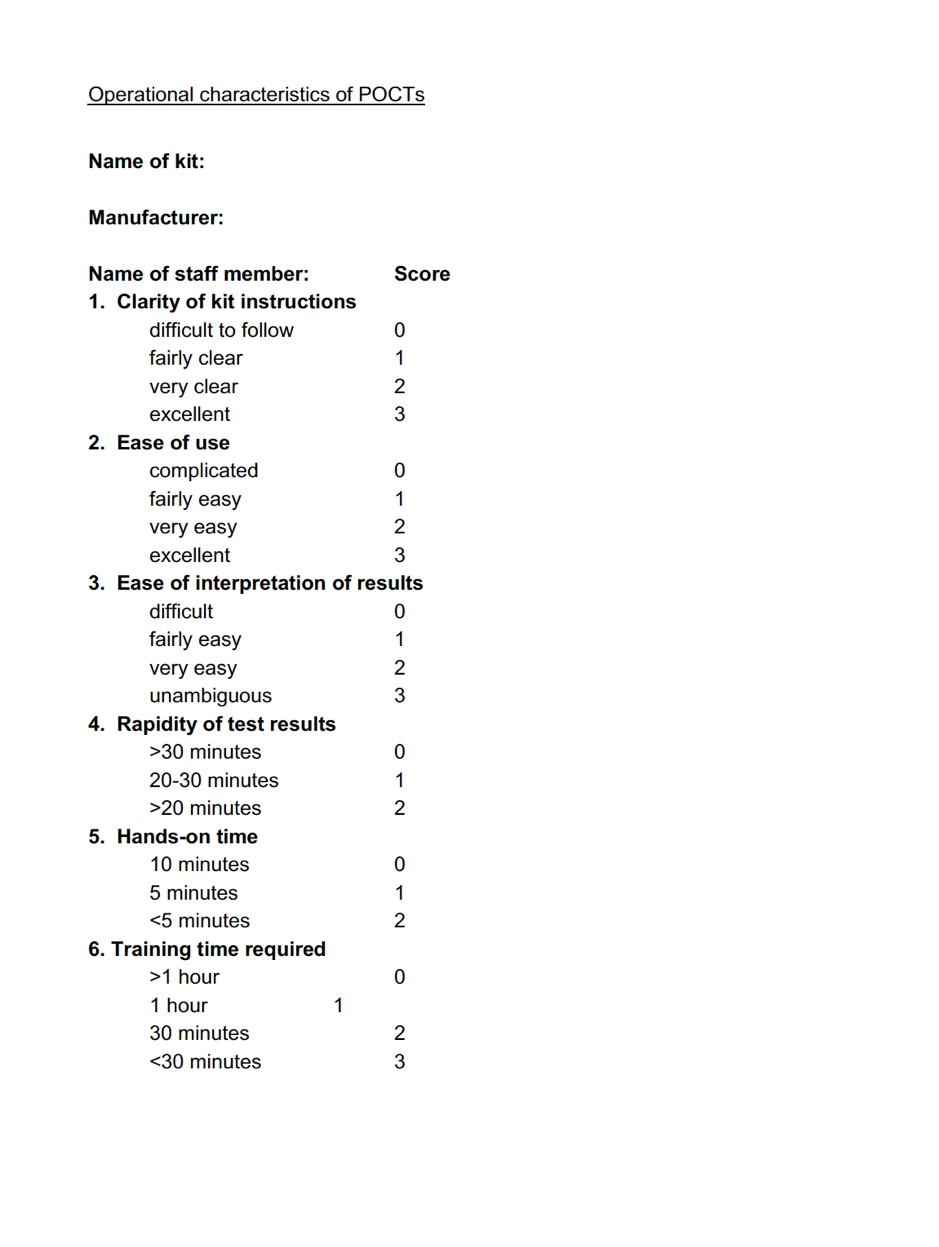

Supplement: S3 Fig — (PNG) [file pone.0198698.s006.png]
