# Supplementary material for: Clinic-based evaluation study of the diagnostic accuracy of a dual rapid test for the screening of HIV and syphilis in pregnant women in Nigeria
Source: PLoS One. 2018 Jul 10;13(7):e0198698. doi: 10.1371/journal.pone.0198698 (PMC6038984; doi:10.1371/journal.pone.0198698)
Supplement: S1 File — (DOCX) [file pone.0198698.s007.docx]

|  | **Section & Topic** | **No** | **Item** | **Reported on page #** |
| --- | --- | --- | --- | --- |
|  |  |  |  |  |
|  | **TITLE OR ABSTRACT** |  |  |  |
|  |  | **1** | Identification as a study of diagnostic accuracy using at least one measure of accuracy  (such as sensitivity, specificity, predictive values, or AUC) | P1: Clinic-based evaluation study of the diagnostic accuracy of a dual rapid test for the screening of HIV and syphilis in pregnant women in Nigeria  P2: Sensitivity and specificity of the HIV component of the dual RDT were 100.0% (95% CI 100.0-100.0) and 99.9% (95% CI 99.8-100.0) respectively, when compared with the national rapid testing algorithm... The specificity of the syphilis component of the dual RDT was 99.9% (95% CI 99.8 – 100.0). |
|  | **ABSTRACT** |  |  |  |
|  |  | **2** | Structured summary of study design, methods, results, and conclusions  (for specific guidance, see STARD for Abstracts) | P2 - 3:  Background  Screening pregnant women for HIV and syphilis is recommended by WHO in order to reduce mother-to-child transmission. We evaluated the field performance and acceptability of a dual rapid diagnostic test (RDT) for HIV and syphilis test in antenatal clinic settings in Nigeria.  Methods and Findings  Participants were recruited at 12 antenatal clinic sites in three states of Nigeria. All consenting individuals were tested according to the national HIV testing algorithm, as well as a dual RDT, the SD BIOLINE HIV/Syphilis Duo Test (Alere, USA), in the clinic. To determine sensitivity, specificity, and concordance, whole blood samples were obtained for repeat RDT performance in the laboratory, as well as reference tests for HIV and syphilis. Dual test acceptability and operational characteristics were assessed among participants and clinic staff.  The prevalence of HIV among the 4,551 enrollees was 3.0% (138/4551) using the national clinic-based HIV testing algorithm. Sensitivity and specificity of the HIV component of the dual RDT were 100.0% (95% CI 100.0-100.0) and 99.9% (95% CI 99.8-100.0) respectively, when compared with the national rapid testing algorithm. The prevalence of syphilis, using TPHA as the reference test, was low at 0.09% (4/4550). The sensitivity of the syphilis component of the dual RDT could not be calculated as no positive results were observed for patients that were positive for syphilis by TPHA. Each of the only four TPHA-positive specimens had RPR titers of 1:1 (neat), indicative of non-active syphilis. The specificity of the syphilis component of the dual RDT was 99.9%  (95% CI 99.8 – 100.0).  The dual RDT received moderately favorable feasibility ratings among antenatal care clinic staff. Acceptability among study participants was high with most women reporting preference for rapid dual HIV/syphilis testing.  Conclusions  The SD BIOLINE HIV/Syphilis Duo Test showed a high overall diagnostic accuracy for HIV and a high specificity for syphilis diagnosis in antenatal clinic settings. This study adds to a growing body of evidence that supports the clinic-based use of dual tests for HIV and syphilis among pregnant women. |
|  | **INTRODUCTION** |  |  |  |
|  |  | **3** | Scientific and clinical background, including the intended use and clinical role of the index test | P4:  Describes clinical importance of screening pregnant women for HIV and syphilis, with particular focus on the study setting.  P5:  Introduces dual HIV/syphilis rapid diagnostic tests (RDTs), their role and intended use:  RDTs … may improve the timely detection of and treatment of HIV and syphilis infections among pregnant women in developing countries, which will facilitate elimination of mother-to-child transmission of HIV and syphilis when employed in antenatal settings [12, 13]. Recently, dual RDTs, used at the point-of-care for simultaneously detecting antibodies to HIV and Treponema pallidum, the causative agent of syphilis (dual HIV & syphilis treponemal RDTs) using venous whole blood, serum/plasma, or finger-stick whole blood have been developed and are now commercially available [13] [14] [15]. To date, there are few data on the performance of these dual RDTs in ANC settings, although they have been evaluated in several laboratory-based and field-based studies and have shown encouraging sensitivities and specificities as compared with reference laboratory tests. |
|  |  | **4** | Study objectives and hypotheses | P6:  The primary objective of this study was to determine the diagnostic accuracy performance of the SD BIOLINE HIV/Syphilis Duo Test for the screening of HIV and syphilis in pregnant women compared to that of (1) the national HIV testing algorithm and (2) a laboratory-based HIV Enzyme Immunoassay (EIA) as two reference standards for HIV, and the T. pallidum Hemagglutination assay (TPHA) as the syphilis reference standard. The secondary objective was to assess the acceptability and operational characteristics of the dual test among pregnant women and antenatal care clinic staff. |
|  | **METHODS** |  |  |  |
|  | *Study design* | **5** | Whether data collection was planned before the index test and reference standard  were performed (prospective study) or after (retrospective study) | P6-7:  A rapid assessment of facilities was conducted to assess and select suitable sites for this prospective study, and to identify study teams from each selected clinic facility. The selection criteria for the study sites included: (1) access to a sufficiently large population of pregnant women attending their first ANC visit in order to complete patient recruitment within 12 weeks of study initiation; (2) close proximity to the reference laboratory so that whole blood for reference tests could be processed and stored within eight hours of collection; (3) estimated prevalence of HIV and syphilis prevalence sufficiently high to achieve study parameters for performance analysis. Three reference laboratories and 12 enrollment sites were selected in three states (Oyo, Imo and the Federal Capitol Territory (FCT)) in Nigeria. The sites chosen were healthcare facilities providing ANC services including HIV and syphilis testing.  The selection criteria for the reference laboratories selected included: (1) routine availability of reference testing for HIV and syphilis; (2) evidence of ongoing accreditation for laboratory quality assurance and management systems; (3) sufficiently trained laboratory and site staff with capacity to perform the study in accordance with the study protocol; (4) strong interest and commitment to research. Study teams consisted of nurses, physicians, laboratory scientists, phlebotomists, and record officers. Supervisory level staff included the state and site coordinator, data managers and reference laboratory coordinators.  Pre-enrollment study activities and staff training  Staff training was conducted over two seven-hour training sessions that took place over two consecutive days in each of FCT, Oyo and Imo states prior to study initiation. S1 Fig shows an example training agenda. All study staff attended these trainings (S1 Table).  At least five staff members from each facility were trained in the use of the dual RDT. These included the site coordinator, site ANC nurses, the site laboratory phlebotomist, and the site record officer. Based on a simple training module, a pictorial training chart appropriate for the local cultural context was developed and used to train on the method to perform the tests safely and accurately and to record results. Hands-on training was provided by the dual RDT manufacturing company to all study staff who would be performing the tests in the field and in the laboratory setting. Training on data collection forms, which included socio-demographic and clinical information on study participants, was provided by national study staff. |
|  | *Participants* | **6** | Eligibility criteria | P7:  Study participants: inclusion and exclusion criteria  Eligible participants included pregnant women presenting for their first ANC visit at one of the 12 study sites. Women were excluded if they were younger than 18 years old or could not provide informed consent. |
|  |  | **7** | On what basis potentially eligible participants were identified  (such as symptoms, results from previous tests, inclusion in registry) | P7:  Study participants: inclusion and exclusion criteria  Eligible participants included pregnant women presenting for their first ANC visit at one of the 12 study sites. Women were excluded if they were younger than 18 years old or could not provide informed consent. |
|  |  | **8** | Where and when potentially eligible participants were identified (setting, location and dates) | Setting/location:  P1 of SI:  S1 Table. Study enrollment sites consisted of 12 ANCs in three states in Nigeria.   \| State \| ANCs \| \| --- \| --- \| \| Federal Capital Territory (FCT) \| Maitama District Hospital  Wuse District Hospital  Asokoro District Hospital  Primary Health Center, Mpape \| \| Ibadan \| State Hospital Adeoyo  Abongbon Primary Health Center  University College Hospital (UCH), Ibadan  Eleta Hospital \| \| Imo \| Federal Medical Center, Owerri  Specialist Hospital Owerri  St David’s Hospital, Owerri  Holy Rosary Hospital, Emekuku \|   Dates:  P8:  Fig 1. Recruitment and enrollment process of pregnant women in ANC clinics.  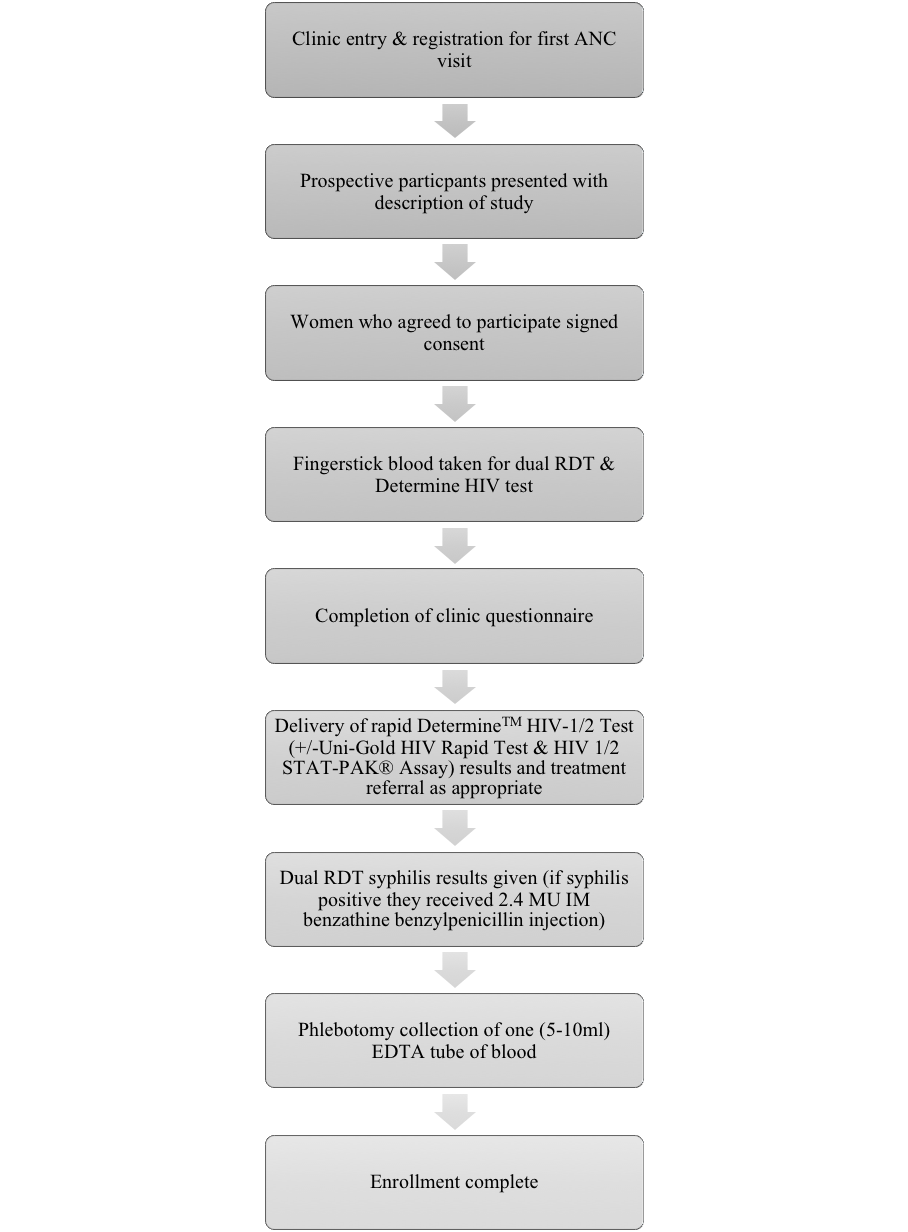 |
|  |  | **9** | Whether participants formed a consecutive, random or convenience series | Convenience series used: P21 |
|  | *Test methods* | **10a** | Index test, in sufficient detail to allow replication | P10:  The fingerprick blood sample obtained was used to perform the SD BIOLINE HIV/Syphilis Duo Test according to the manufacturer’s instructions, as well as the national HIV testing algorithm with Determine^TM^ HIV-1/2 Test (Alere), confirmed by Uni-Gold HIV Rapid Test (Trinity Biotech, Wicklow, Ireland). After 15-20 minutes, the nurse informed participants of the result of the Determine^TM^ HIV-1/2 Test and the syphilis result of the dual RDT and recorded all results on the socio-demographic questionnaire. |
|  |  | **10b** | Reference standard, in sufficient detail to allow replication | P10:  5ml of venipuncture whole blood was collected into an EDTA plasma collection tube for transportation to the lab within eight hours of specimen collection. Specimens were stored at 15°C prior to, and during, transportation to the reference laboratory.  Whole blood samples received in the laboratory were tested with a second dual RDT by laboratory staff who had no access to clinic information of the participants, and no knowledge of the results of the tests carried out in the clinic (Fig 2). For syphilis reference testing, fresh whole blood specimens were tested with the Treponema pallidum hemaggluttination assay (TPHA). Those specimens that tested positive by TPHA were then tested using a quantitative rapid plasma reagin (RPR) and the titer was recorded. The time interval between conducting the dual RDT and the TPHA was a maximum of one week. Remaining specimens were centrifuged and two (2ml) aliquots of plasma were pipetted into cryovials and stored in a -70 C freezer. HIV reference tests were completed using batched samples at the end of the study using the Genscreen Ultra HIV Ag-Ab Assay (BIO-RAD Europe GmbH), a 4th generation HIV test. Frozen cryovials were shipped on dry ice from Imo and FCT states to the reference lab in Oyo state for performance of the Genscreen Ultra HIV Ag-Ab test. The time interval between the field-performed HIV tests and the Genscreen Ultra HIV Ag-Ab Assay was up to four months, as serum was stored (-70C) and batch tested at the end of the study. However, for a given participant, all reference samples in the study were taken at the same time as the field-performed dual RDT. All test results were recorded in a laboratory log. |
|  |  | **11** | Rationale for choosing the reference standard (if alternatives exist) | P6:  The primary objective of this study was to determine the diagnostic accuracy performance of the SD BIOLINE HIV/Syphilis Duo Test for the screening of HIV and syphilis in pregnant women compared to that of (1) the national HIV testing algorithm and (2) a laboratory-based HIV Enzyme Immunoassay (EIA) as two reference standards for HIV, and the *T. pallidum* Hemagglutination assay (TPHA) as the syphilis reference standard.  P13:  The sensitivity and specificity of the dual RDT was assessed using two separate reference standards for HIV; (1) the national rapid testing algorithm with DetermineTM HIV-1/2 Test (Alere) positivity confirmed by a positive Uni-Gold HIV Rapid Test (Trinity Biotech) and (2) the laboratory-based HIV 4th generation Ag/Ab Enzyme Immunoassay (EIA) (Genscreen ULTRA HIV Ag-Ab Assay). This allowed a comparison between the dual RDT and the current HIV testing standard in ANC settings, and also a gold standard laboratory-based assay. The accuracy of the syphilis component was assessed using the TPHA (Human Gesellschaft fur Biochemica und Diagnostica mbH, Wiesbaden, Germany) as a laboratory reference standard for antibodies to T. pallidum. A rapid plasma reagin (RPR) assay (Syphilis RPR test, Human Gesellschaft fur Biochemica und Diagnostica mbH, Wiesbaden, Germany) was conducted for samples that gave a positive TPHA result in order to identify acute infection (RPR> 1:4). |
|  |  | **12a** | Definition of and rationale for test positivity cut-offs or result categories  of the index test, distinguishing pre-specified from exploratory | RDTs were conducted according to the manufacturer’s protocol and results were read by eye. |
|  |  | **12b** | Definition of and rationale for test positivity cut-offs or result categories  of the reference standard, distinguishing pre-specified from exploratory | All laboratory assays were conducted according to the manufactuer’s protocols.  TPHA  (http://www.human-de.com/data/gb/vr/lx-tpha.pdf):  A compact button at the bottom of the wells indicates a negative result cells, while an even layer of agglutinated cells, sometimes surrounded by a more a less distinct ring, indicate a positive result. With the control cells always a well-defined button should be seen.  Genscreen HIV EIA  (http://www.bio-rad.com/webroot/web/pdf/inserts/CDG/en/883605_EN.pdf):  The absorbance of each negative control (R3) should be less than 0.170 : OD R3 < 0.170  If one negative control does not respect this norm, disregard and recalculate the mean using the two remaining values. Only one value may be eliminated by this way  The mean of the absorbance of the negative controls (R3) should be less than 0.150 : OD R3 < 0.150  The absorbance of HIV Ab positive control (R4) should be greater than 0.9 : OD R4 > 0.9  The absorbance of HIV Ag positive control (R5) should be greater than 0.9 : OD R5 > 0.9 |
|  |  | **13a** | Whether clinical information and reference standard results were available  to the performers/readers of the index test | Only basic socio-demographic data was available to the performers/readers of the index test, including age of participant, gestational length and length of travel-to-clinic time.  P10:  All consenting ANC attendees were enrolled by a trained study nurse. The fingerprick blood sample obtained was used to perform the SD BIOLINE HIV/Syphilis Duo Test according to the manufacturer’s instructions, as well as the national HIV testing algorithm with Determine^TM^ HIV-1/2 Test (Alere), confirmed by Uni-Gold HIV Rapid Test (Trinity Biotech, Wicklow, Ireland). After 15-20 minutes, the nurse informed participants of the result of the Determine^TM^ HIV-1/2 Test and the syphilis result of the dual RDT and recorded all results on the socio-demographic questionnaire. |
|  |  | **13b** | Whether clinical information and index test results were available  to the assessors of the reference standard | P10:  Whole blood samples received in the laboratory were tested with a second dual RDT by laboratory staff who had no access to clinic information of the participants, and no knowledge of the results of the tests carried out in the clinic (Fig 2). |
|  | *Analysis* | **14** | Methods for estimating or comparing measures of diagnostic accuracy | P13:  The sensitivity, specificity and kappa statistics [16], as well as their associated 95% confidence intervals, were calculated using the EpiBasix package [17] in R statistics (version 3.4.0) [18]. |
|  |  | **15** | How indeterminate index test or reference standard results were handled | P14:  There were no indeterminate or inconclusive results for any of the RDTs or laboratory assays used in the study. |
|  |  | **16** | How missing data on the index test and reference standard were handled | P14:  Serum samples used for laboratory testing with the dual RDT and reference tests for antibodies to HIV and syphilis were collected from 4,550 participants. One participant experienced failed attempts at blood draw and was therefore unable to be tested using the HIV EIA reference testing. However, rapid test results for the dual RDT and Determine^TM^ HIV-1/2 Test/Uni-Gold™ HIV Rapid Test were available from the ANC setting. Therefore, this individual could only be included in the analysis comparing the accuracy of the HIV component of the dual RDT to Determine^TM^ HIV-1/2 Test/Uni-Gold™ HIV Rapid Test. There were no other missing results… |
|  |  | **17** | Any analyses of variability in diagnostic accuracy, distinguishing pre-specified from exploratory | N/A |
|  |  | **18** | Intended sample size and how it was determined | P8:  Sample size calculations for operational performance assessment  The sample size calculation was based on the laboratory performance of the dual RDT compared to the reference standard and the estimated HIV and syphilis prevalence in Nigeria. A disease seroprevalence of 5% for both HIV and syphilis in pregnant women was assumed based on recent surveillance data (2015), an assumed higher risk of HIV and/or syphilis in pregnant women, and the low uptake of HIV and syphilis testing in ANCs in Nigeria. Using an estimated test sensitivity of 98%, an alpha value of 0.05 and a beta value of 80% (power), a sample size of 3,800 was estimated. Based on the impreciseness of the available prevalence rates, particularly for syphilis, the sample size was increased to 4,500 women. |
|  | **RESULTS** |  |  |  |
|  | *Participants* | **19** | Flow of participants, using a diagram | P9  Fig 1. Recruitment and enrollment process of pregnant women in ANC clinics.  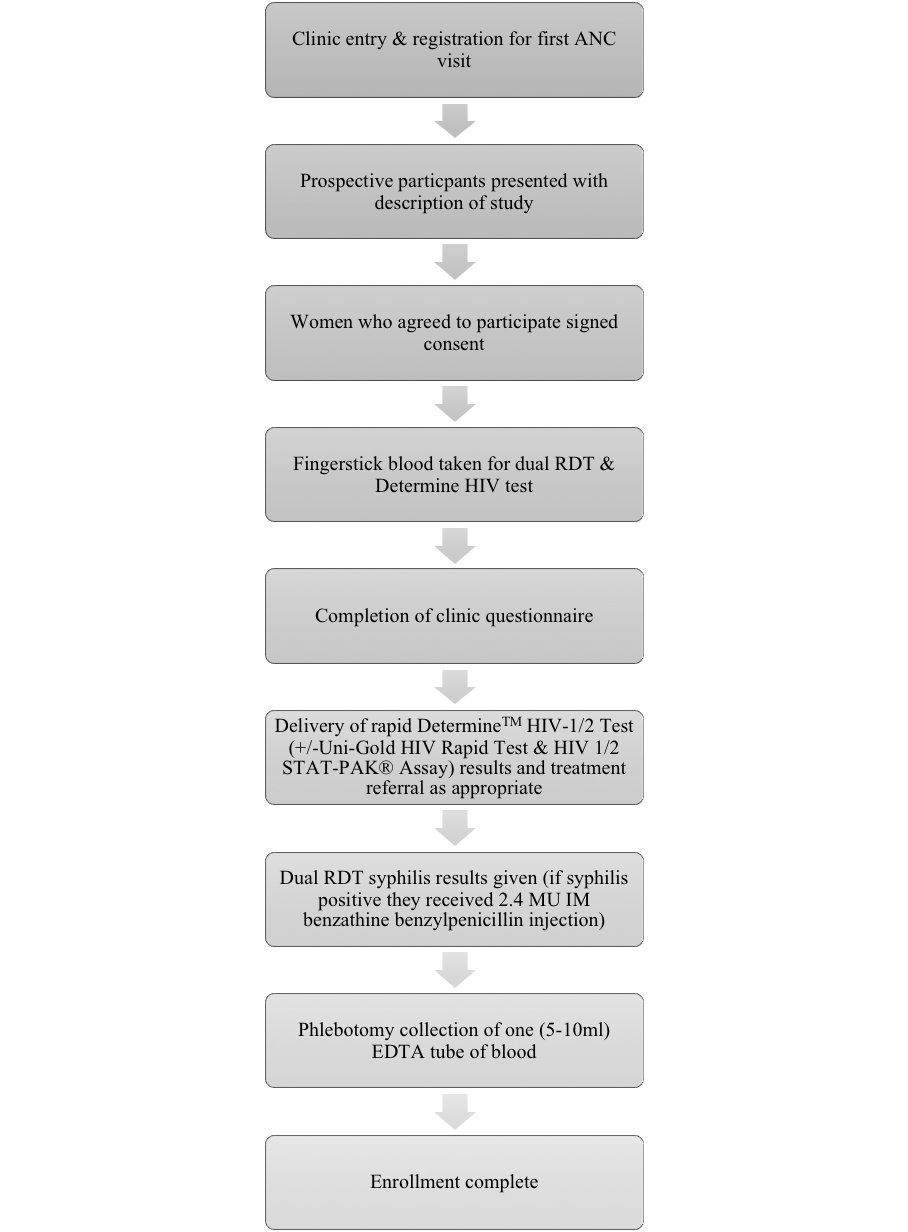 |
|  |  | **20** | Baseline demographic and clinical characteristics of participants | P13:  Study population  Table 1 lists the characteristics of the 4,551 study participants recruited across the 12 selected study sites. The median age of the women recruited to the study was 30 years (Interquartile range (IQR): 27 - 33). The median gestational age was 20 weeks (IQR: 16 - 28) and the median time (minutes) taken to reach the clinic from their homes was 30 minutes (IQR: 30 – 50). |
|  |  | **21a** | Distribution of severity of disease in those with the target condition | ?  HIV – some acute infection, not detected  Syph – some resolved infection |
|  |  | **21b** | Distribution of alternative diagnoses in those without the target condition | N/A |
|  |  | **22** | Time interval and any clinical interventions between index test and reference standard | P10:  For syphilis reference testing, fresh whole blood specimens were tested with the *Treponema pallidum* hemaggluttination assay (TPHA). Those specimens that tested positive by TPHA were then tested using a quantitative rapid plasma reagin (RPR) and the titer was recorded. The time interval between conducting the dual RDT and the TPHA was a maximum of one week. Remaining specimens were centrifuged and two (2ml) aliquots of plasma were pipetted into cryovials and stored in a -70°C freezer. HIV reference tests were completed using batched samples at the end of the study using the Genscreen Ultra HIV Ag-Ab Assay (BIO-RAD Europe GmbH), a 4^th^ generation HIV test. Frozen cryovials were shipped on dry ice from Imo and FCT states to the reference lab in Oyo state for performance of the Genscreen Ultra HIV Ag-Ab test. The time interval between the field-performed HIV tests and the Genscreen Ultra HIV Ag-Ab Assay was up to four months, as serum was stored (-70°C) and batch tested at the end of the study. However, for a given participant, all reference samples in the study were taken at the same time as the field-performed dual RDT. All test results were recorded in a laboratory log.  P11:  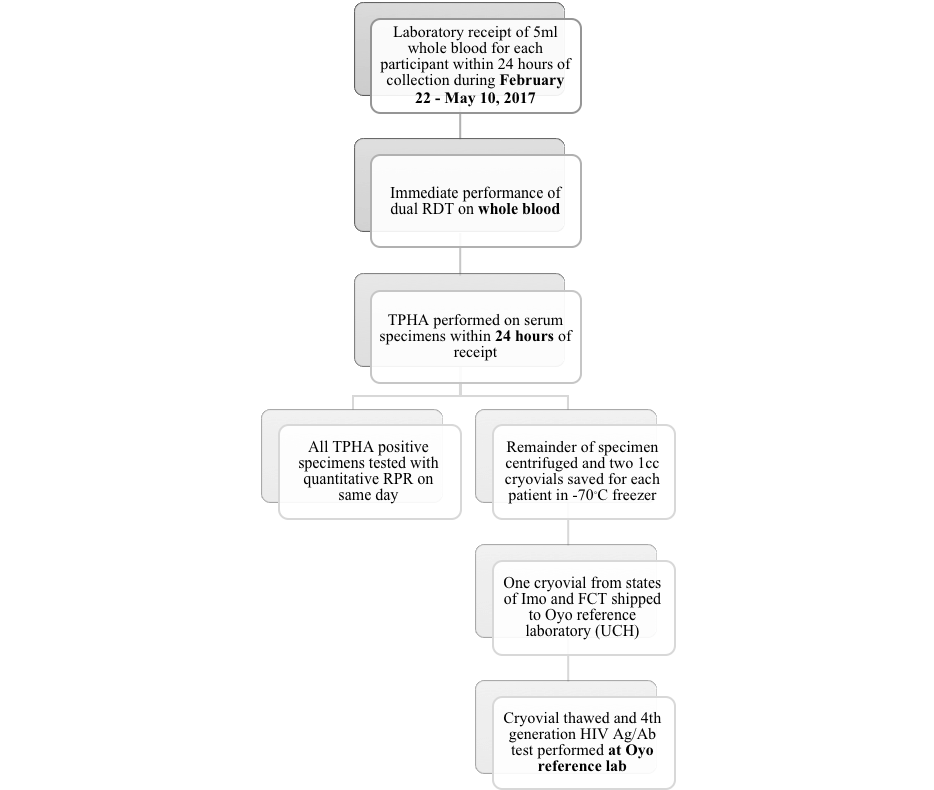 |
|  | *Test results* | **23** | Cross tabulation of the index test results (or their distribution)  by the results of the reference standard | P16:   \| Index test \| Reference test \| TP \| FN \| FP \| TN \| Total \| Sensitivity (%) (95% CI) \| Specificity (%) (95% CI) \| Kappa Coefficient (95% CI) \| \| --- \| --- \| --- \| --- \| --- \| --- \| --- \| --- \| --- \| --- \| \| SD BIOLINE HIV/Syphilis Duo Test (HIV) \| Determine^TM^ HIV-1/2 Test (+/-Uni-Gold HIV Rapid Test & HIV 1/2 STAT-PAK® Assay) \| 138 \| 0 \| 6 \| 4407 \| 4551 \| 100.0 (100.0 – 100.0) \| 99.9 (99.8 – 100.0) \| 0.978 (0.960 – 0.996) \| \| SD BIOLINE HIV/Syphilis Duo Test (HIV) \| Genscreen ULTRA HIV Ag-Ab Assay \| 121 \| 20 \| 22 \| 4387 \| 4550 \| 85.8 (80.1 – 91.6) \| 99.5 (99.3 – 99.7) \| 0.847 (0.802 – 0.893) \| \| Determine^TM^ HIV-1/2 Test (+/-Uni-Gold HIV Rapid Test & HIV 1/2 STAT-PAK® Assay) \| Genscreen ULTRA HIV Ag-Ab Assay \| 115 \| 26 \| 22 \| 4387 \| 4550 \| 81.6 (75.2 – 88.0) \| 99.5 (99.3 – 99.7) \| 0.822 (0.772 – 0.871) \| \| SD BIOLINE HIV/Syphilis Duo Test (syphilis) \| Syphilis TPHA \| 0 \| 4 \| 4 \| 4542 \| 4550 \| N/A \| 99.9 (99.8 – 100) \| N/A \| |
|  |  | **24** | Estimates of diagnostic accuracy and their precision (such as 95% confidence intervals) | P16:   \| Index test \| Reference test \| TP \| FN \| FP \| TN \| Total \| Sensitivity (%) (95% CI) \| Specificity (%) (95% CI) \| Kappa Coefficient (95% CI) \| \| --- \| --- \| --- \| --- \| --- \| --- \| --- \| --- \| --- \| --- \| \| SD BIOLINE HIV/Syphilis Duo Test (HIV) \| Determine^TM^ HIV-1/2 Test (+/-Uni-Gold HIV Rapid Test & HIV 1/2 STAT-PAK® Assay) \| 138 \| 0 \| 6 \| 4407 \| 4551 \| 100.0 (100.0 – 100.0) \| 99.9 (99.8 – 100.0) \| 0.978 (0.960 – 0.996) \| \| SD BIOLINE HIV/Syphilis Duo Test (HIV) \| Genscreen ULTRA HIV Ag-Ab Assay \| 121 \| 20 \| 22 \| 4387 \| 4550 \| 85.8 (80.1 – 91.6) \| 99.5 (99.3 – 99.7) \| 0.847 (0.802 – 0.893) \| \| Determine^TM^ HIV-1/2 Test (+/-Uni-Gold HIV Rapid Test & HIV 1/2 STAT-PAK® Assay) \| Genscreen ULTRA HIV Ag-Ab Assay \| 115 \| 26 \| 22 \| 4387 \| 4550 \| 81.6 (75.2 – 88.0) \| 99.5 (99.3 – 99.7) \| 0.822 (0.772 – 0.871) \| \| SD BIOLINE HIV/Syphilis Duo Test (syphilis) \| Syphilis TPHA \| 0 \| 4 \| 4 \| 4542 \| 4550 \| N/A \| 99.9 (99.8 – 100) \| N/A \| |
|  |  | **25** | Any adverse events from performing the index test or the reference standard | P14:  Two participants received benzathine benzylpenicillin due to a positive syphilis dual RDT result, but subsequently had a negative TPHA result. This minor overtreatment is the only adverse effect of the study that the authors are aware of. |
|  | **DISCUSSION** |  |  |  |
|  |  | **26** | Study limitations, including sources of potential bias, statistical uncertainty, and generalisability | Limitations:  P21:  There are limitations that should be considered regarding the interpretation of these data. First, this was a convenience sample of pregnant women attending their first ANC visit limiting representativeness of pregnant women in Nigeria overall. Participant enrollment was limited to a specific time period reflecting the expiration dates of the donated test kits. The sensitivity of the syphilis component of the dual RDT was a key expected result from the study. However, a low prevalence of syphilis in this large study size of 4,551 pregnant women was unanticipated and prevented this calculation. External quality assurance of HIV and syphilis testing was not performed through an outside reference lab. However, each of the reference laboratories were nationally accredited. Finally, use of the 4^th^ generation HIV Ag/Ag test (Genscreen ULTRA HIV Ag-Ab Assay) as the laboratory reference standard for HIV testing did not reflect a true comparison with the HIV EIA component of the dual RDT. The ability of the 4^th^ generation HIV to detect acute HIV infection prior to antibody seroconversion was reflected in the calculated sensitivities for the dual RDT test as well as the national rapid HIV testing algorithm which uses Determine^TM^ HIV-1/2 Test as the initial screening test.  Bias and statistical uncertainty:  All patients included in the analysis of dual RDT diagnostic accuracy also had a reference test performed – this avoided verification bias.  Selection bias was avoided by using a convenience sample representative of the population the test is intended for. Observer bias was avoided because the laboratory staff had no knowledge of the index test results. Differential reference bias was avoided because all samples were tested using the same reference tests.  P20  Although the sensitivity of the dual RDT was higher for HIV diagnosis than the national HIV testing algorithm, this difference was not statistically significant, as the confidence intervals for each of the sensitivities overlapped.  Generalisability:  P22  Ministries of Health, outside of Nigeria, considering a similar implementation may be interested in the evaluation findings and this approach to validate test performance and assess acceptability, feasibility and dual testing barriers and facilitators within their own countries. This study adds to a growing body of evidence that supports the clinic-based use of dual tests for HIV and syphilis among pregnant women, and can be used as further justification of their use in antenatal settings. |
|  |  | **27** | Implications for practice, including the intended use and clinical role of the index test | P21-22  The results from this assessment are being used by the Nigerian MoH to facilitate initial decision-making for future use and potential scale-up of the dual test in ANC settings. As the Nigerian MoH reviews these favorable outcomes, the next step potentially is how this information will be used to inform national policies, HIV and syphilis testing algorithms, and clinic practices of the sites included in the evaluation. An in-depth qualitative assessment may help the MoH identify ways to address barriers and implement strategies to improve acceptability and feasibility of dual HIV/syphilis testing provision and develop a plan of action to address key barriers.  Ministries of Health, outside of Nigeria, considering a similar implementation may be interested in the evaluation findings and this approach to validate test performance and assess acceptability, feasibility and dual testing barriers and facilitators within their own countries. This study adds to a growing body of evidence that supports the clinic-based use of dual tests for HIV and syphilis among pregnant women, and can be used as further justification of their use in antenatal settings. |
|  | **OTHER INFORMATION** |  |  |  |
|  |  | **28** | Registration number and name of registry | N/A |
|  |  | **29** | Where the full study protocol can be accessed | N/A |
|  |  | **30** | Sources of funding and other support; role of funders | P23:  This work was funded by the UNDP-UNFPA-UNICEF-WHO-World Bank Special Programme of Research, Development and Research Training in Human Reproduction (HRP), a cosponsored program executed by the World Health Organization (WHO). |
|  |  |  |  |  |
